# Proteomic analysis of neonatal mouse hearts shows PKA functions as a cardiomyocyte replication regulator

**DOI:** 10.1186/s12953-023-00219-4

**Published:** 2023-10-11

**Authors:** Lizhi Hu, Minglu Liang, Qin Jiang, Youming Jie, Long Chen, Fengxiao Zhang

**Affiliations:** 1 Department of Cardiology, Union Hospital, Tongji Medical College, Huazhong University of Science and Technology, Wuhan, 430022 China; 2 Clinic Center of Human Gene Research, Union Hospital, Tongji Medical College, Huazhong University of Science and Technology, Wuhan, China; 3 Department of Cardiovascular Diseases, Liyuan Hospital, Tongji Medical College, Huazhong University of Science and Technology, Wuhan, China

**Keywords:** Proteomics, Protein kinase A, Myocardial regeneration

## Abstract

The ability of the adult mammalian heart to regenerate can save the cardiac muscle from a loss of function caused by injury. Cardiomyocyte regeneration is a key aspect of research for the treatment of cardiovascular diseases. The mouse heart shows temporary regeneration in the first week after birth; thus, the newborn mouse heart is an ideal model to study heart muscle regeneration. In this study, proteomic analysis was used to investigate the differences in protein expression in the hearts of neonatal mice at days 1 (P1 group), 4 (P4 group), and 7 (P7 group). Kyoto Encyclopedia of Genes and Genomes pathway enrichment analysis showed changes in several groups of proteins, including the protein kinase A (PKA) signaling pathway. Moreover, it was found that PKA inhibitors and agonists regulated cardiomyocyte replication in neonatal mouse hearts. These findings suggest that PKA may be a target for the regulation of the cardiomyocyte cell cycle.

**Supplementary Information:**

The online version contains supplementary material available at 10.1186/s12953-023-00219-4.

## Introduction

Cardiomyocytes are terminally differentiated cells with very limited regenerative capacity. Several sources of injury can cause irreversible myocardial cell death, which results in the permanent loss of cardiac function and subsequently leads to cardiac diseases including myocardial infarction, malignant arrhythmias, heart failure, and sudden cardiac death [1]. Heart injury is usually associated with cardiomyocyte death and a good understanding of the ability of cardiomyocyte replication to repair the injured heart is lacking. Cardiac fibroblast proliferation and scar formation can lead to cardiac insufficiency, arrhythmias, and eventual death [2]. Cardiomyocyte death and diminished pump function are difficult to reverse in adults. Scientists worldwide have focused on cardiac regeneration for the replacement of dead and damaged cardiomyocytes with new cells to improve heart function [3–6].

The rate of cardiomyocyte renewal decreases with age and less than half of cardiomyocytes are renewed during the normal human lifespan [7]. The regenerative ability in mammals is based on myocardial cell proliferation in the early stages after birth and is strong enough to repair the damaged heart tissue and restore the normal function of the heart [8]. However, this ability reduces with age and is extremely rare in adults. Therefore, researchers have developed many stem cell therapies that target the damaged myocardium. It has been shown that embryonic stem cells, pluripotent stem cells, bone marrow-derived stem cells-derived epicardial cells, and cardiomyocytes increased cardiomyocyte proliferation, and improved cardiac function [9–11]. Moreover, it is known that cardiomyocytes in adults can dedifferentiate under certain conditions or regress to similar early stages of development to enable regeneration. Activation of the expression of certain genes that are normally only expressed during early development of the heart may improve dedifferentiation [12].

Protein kinase A (PKA) is a cAMP-dependent kinase. Once activated, it can phosphorylate various proteins and regulate cellular functions, including gene transcription, energy metabolism, cell cycle, and apoptosis, in almost all mammalian tissues [13]. The PKA signaling system includes both regulatory and catalytic components. The regulatory components are receptors for cAMP and are encoded by four genes (RIα, RIβ, RIIα, and RIIβ). While these four regulatory isoforms are functionally nonredundant and tissue-specific, the catalytic components are similar [14]. The catalytic components perform the kinase function and have three isoforms (C-α, C-β, and C-γ). In the presence of cAMP, the catalytic components dissociate from the regulatory components and phosphorylate substrates [15].

A previous study has confirmed that PKA overactivation leads to heart failure [16]. In mice, PKA expression in the myocardium increases after myocardial infarction [17]. Furthermore, PKA knockout can protect mice from age-related degeneration of cardiac function [18]. However, the role of PKA in cardiomyocyte replication has not been confirmed.

In this study, we compared proteomic changes in newborn mouse hearts over time and found that the PKA signaling pathway is a target in regulating the cardiomyocyte cell cycle. To investigate whether the PKA signaling pathway regulated cardiomyocyte regeneration, we used a PKA inhibitor and agonist to treat primary cardiomyocytes. Our findings suggested that PKA activity was closely related to cardiomyocyte replication. Treatment with the PKA inhibitor could promote cardiomyocyte division, whereas treatment with the PKA agonist inhibited cardiomyocyte entry into the cell cycle.

## Materials and methods

### Animals

Neonatal C57BL/6 mice from specific families (P1, P4, and P7) were euthanized with pentobarbital and their hearts were excised for protein extraction.

### Protein extraction, digestion, and liquid chromatography with tandem mass spectrometry (LC–MS/MS)

Ventricular tissue samples from the P1 (*n* = 4), P4 (*n* = 3), and P7 (*n* = 3) groups were homogenized in RIPA lysis buffer containing a protease inhibitor cocktail (Roche). The homogenates were centrifuged, and the Bradford assay was used to measure the protein concentration in the supernatant. To minimize biological variations, we used a pooled approach to extract protein. Equal weight ventricular tissues from the same day (day 1 *n* = 4, day 4 *n* = 3, day 7 *n* = 3) were pooled together as described in previous studies [19, 20], and then homogenized in RIPA lysis buffer containing a protease inhibitor cocktail.

From each group, 500 µg of the protein from the solution digest was used according to the method described previously [21]. The protein in the supernatant was precipitated and centrifuged. The pellets were resuspended and reduced with DTT and then alkylated using iodoacetamide. Lastly, trypsin (Promega) was added to the protein solution in a 1:50 ratio (trypsin/protein, w/w) and incubated at 37°C for 12 h.

Stable isotope dimethyl labeling, a reliable, cost-effective, and undemanding procedure method was used to relative quantification. Dimethyl labeling was performed as previously described with minor modifications [22]. Briefly, P1 samples were labeled with 4% CH2O and NaBH3CN (light labeling), P4 samples were labeled with 4% CD2O and NaBH3CN (middle labeling), and P7 samples were labeled with 4% 13CD2O and NaBD3CN (heavy labeling). Equal amounts of peptides from the light-, middle-, and heavy-labeled samples were mixed and desalted using C18 columns (Waters). Strong cation–exchange chromatography was used to separate the mixed samples. Eight fractions were collected and desalted using gradient elution before MS analysis. MS/MS analysis was performed using a QE-HF mass spectrometer (Thermo Fisher Scientific) coupled with a nano-HPLC system (SCIEX). The suspended peptides were first loaded onto a C18 trap column (0.5 mm × 2 mm, MICHROM Bioresources, Inc.) and eluted from the trap column to a C18 analytical column (100 μm × 150 mm, 100 Å pore size, 3 μm particle size, MICHROM Bioresources, Inc.) at a flow rate of 300 nL/min. Liquid chromatography was performed using a 100-min gradient (mobile phase A: 3% DMSO, 97% H2O, 0.1% formic acid; mobile phase B: 3% DMSO, 97% ACN, 0.1% formic acid; gradient: 0 min in 5% B, 65 min of 5–23% B, 20 min of 23–52% B, 1 min of 52–80% B, 80% B for 4 min, 0.1 min of 80–5% B, 5% B for 9.9 min). Proteome Discoverer Software Version 2.1 (PD2.1, Thermo Scientific) was used for the relative quantification of proteins. The UniProt database (Mus musculus.20160318.fasta, Proteome ID: UP000000589, Download date: 2016 Mar 18) was used. Parameters were set as follows: parent ion tolerance 10 ppm; fragment ion mass tolerance 0.02 Da; carbamidomethyl cysteine as a fixed modification; methionine oxidation and dimethylation 3plex (C2H4, C2D4, 13C2D4) were variable modifications. The peptide charge was set to 2 + or 3 + , and up to two missed cleavages were allowed. The proteins and peptides denoted with high FDR confidence (FDR < 1%).

### Bioinformatics analysis

The threshold of “significantly changed” ratios was set by analyzing the Gaussian distribution of the dimethyl-labeling ratio (logarithmized to base 2). To place a threshold at the 95% confidence limit (*p* ≤ 0.05), we set mean ± 1.96 standard deviations as the “significantly changed” threshold [23]. Protein ratios were uploaded to the PANTHER website (http://www.pantherdb.org/) for statistical enrichment, which can identify specific protein classes that are significantly changed during the development of the heart. Intracellular pathway analysis was performed using the Kyoto Encyclopedia of Genes and Genomes (KEGG) pathways database. Significantly changed KEGG pathways were identified based on the hypergeometric distribution of genes with *p* < 0.05.

### Cell culture

Briefly, one-day-old C57BL/6 mice were anesthetized with pentobarbital and were subjected to thoracotomy to expose the heart. Hearts were quickly isolated and cardiomyocyte isolation and cell culture were performed following a previously reported protocol with slight modifications [24]. Cardiomyocytes were cultured for future experiments.

### 5-Ethynyl-2′-deoxyuridine (EdU) incorporation assay

EdU assays were used to determine cardiomyocyte replication. Neonatal mouse cardiomyocytes were treated with the vehicle and the PKA agonist bucladesine (MedChemExpress, HY-15979) or the PKA inhibitor H-89 (MedChemExpress, HY-15979). Six hours later, EdU (50 μM) was added to cardiomyocytes and cultured for 24 h. EdU staining was conducted using the Cell-Light EdU Apollo567 in vitro kit according to the manufacturer’s instructions (RIBOBIO, C10310-1). Cardiomyocytes were stained with an anti-cTnT antibody (Abcam catalog no. ab8295) combined with Alexa Fluor® 488 conjugate second antibody (CST catalog no. 4480). Next, 4′,6-diamidino-2-phenylindole (DAPI) counterstaining (Vector lab, H1200) was performed. Stained sections were photographed using a Zeiss microscope and EdU-positive cardiomyocytes (red, green, and blue merge) were quantified using ImageJ software (3 random fields for each well, 3 replicate wells for each experiment). Three independent experiments were used for data analysis.

### Ki67 detection

Neonatal mouse cardiomyocytes were treated with the drugs as described above. Ki67 was stained using the anti-Ki67 antibody (Abcam catalog no. ab15580) combined with Alexa Fluor® 594 Conjugate (CST catalog no. 8889). Cardiomyocytes and nuclei were stained with cTnT and DAPI as described above. Ki67-positive cardiomyocytes (red, green, and blue merge) were quantified using ImageJ software (3 random fields for each well, 3 replicate wells for each experiment). Three independent experiments were used for data analysis.

### RT-PCR assays

Ventricular tissue samples from neonatal mice were homogenized in TRIzol reagent (Invitrogen) and the total RNA was extracted according to the manufacturer’s protocols. One μg of total RNA (1 mg) was used for reverse transcription (TaKaRa). qRT-PCR was carried out in a 10-μL reaction volume containing SYBR Green Master Mix (TaKaRa) and primers. The sequences of PCR primer are shown as follows: Ctbp2 5′- AGTCTCCACACACCTCATTCA-3′, and 5′-GGGGCGGATACTTGGTTC-3′; Grb10 5′– CACCTGCCAAGCATTTCCCT-3′, and 5′-GTGTCGGTTGGACACTGGTT-3′; Erbin 5′- GCACATTTTTCGACATCCGCA-3′, and 5′-CAGGCTGTCTGGAAGACCTC-3′; Pkn2 5′- CACCCGTTTTTCCGGCTAAC-3′, and 5′-TGGTTCTCGAGGTGGAGTCA-3′; Tsfm 5′- GGCCCTAGCGATTGGTAAACT-3′, and 5′-GCCGACATAGAACCCAGAGG-3′; Stat3 5′- GCCCCGTACCTGAAGACCAA-3′, and 5′-ACGTGAGCGACTCAAACTGC-3′; Acox1 5′- CGTCGAGAAATCGAGAACTTG-3′, and 5′-GGTTCCACAAAATTGACCATATGTA-3′; Lipe 5′- CCAGGGAGGGCCTCAGC-3′, and 5′-TGTCTTCTGCGAGTGTCACC-3′; Adcy5 5′- TGGTGGACCGTGTTCTTCATC-3′, and 5′-ATGAGGACATTGGAGACAAGCTGTT-3′; Acox3 5′- GACAAAGCAGGTCGGTGACA-3′, and 5′- CAGCTCCCCAGAGTTGAAGG-3’, GAPDH 5′- AATGGGCAGCCGTTAGGAAA-3′, and 5′- GCCCAATACGACCAAATCAGAG-3′.

### Preparation of whole-cell extracts and western blotting

Whole-cell extracts were prepared, and western blotting was conducted as described previously [25]. Briefly, ventricular tissue samples from neonatal mice or the neonatal cardiomyocytes were homogenized in RIPA lysis buffer (Thermo Scientific™) containing a protease inhibitor cocktail and phosphatase inhibitor PhosStop (Roche). The homogenates were centrifuged at 12 000 x*g* for 15 min and protein concentrations were determined using a BCA protein assay kit (Pierce). Proteins were normalized to the same amount and treated with SDS-PAGE loading buffer. Then the proteins were separated using SDS-PAGE and electrotransferred onto a nitrocellulose membrane (Bio-Rad). Antibodies against PKA α/β/γ pT197 (Abcam catalog no. ab75991), PKA α/β/γ (Santa Cruz catalog no. sc-365615), CDK4 (ABclonal catalog no. A11136), cyclin D1 (ABclonal catalog no. A19038), cyclin E1 (ABclonal catalog no. A12000), Rb (bimake.cn, catalog no. A5073), phosph-Rb(s807) antibody (bimake.cn, catalog no. A5735), Ctbp2 (Proteintech Cat No. 10346–1-AP), Grb10 (Proteintech Cat No. 23591–1-AP), Stat3 (Proteintech Cat No. 10253–2-AP) and GAPDH (ABclonal catalog no. A19056) were used as primary antibodies. Protein signals were determined using an electrochemiluminescence western blotting substrate (Pierce) and analyzed using Image Lab software (version 5.2.1, Bio-Rad).

### Statistical enrichment test

The statistical enrichment test is a statistical analysis tool offered by the PANTHER website (http://www.pantherdb.org/) [26]. The submitted proteins were grouped according to the GO or PANTHER classification, and the expression ratio of a particular group of proteins was compared with that of all proteins in the list to assess the likelihood of a significant change in a particular group of proteins.

### Pathway analysis

The KEGG pathway database was used for pathway research (http://www.kegg.jp/). This analysis divides proteins in the KEGG pathway and sets all qualitative proteins as the background. The KEGG pathway analysis is based on supergeometric testing and reveals protein enrichment levels of each pathway to determine significant impacts of metabolism and signal transduction pathways.

### Statistical analysis

All statistical analyses were performed using SPSS software (version 20.0, IBM Corp., USA). Values are presented as mean ± standard error of the mean (SEM [*n* ≥ 3]). *P* < 0.05 was considered significant. The significance of differences was determined using independent samples *t*-tests or one-way analysis of variance followed by Student-Newmann-Keuls multiple comparison tests.

## Results

### Age-related proteomic changes in mouse hearts

**Fig. 1:**
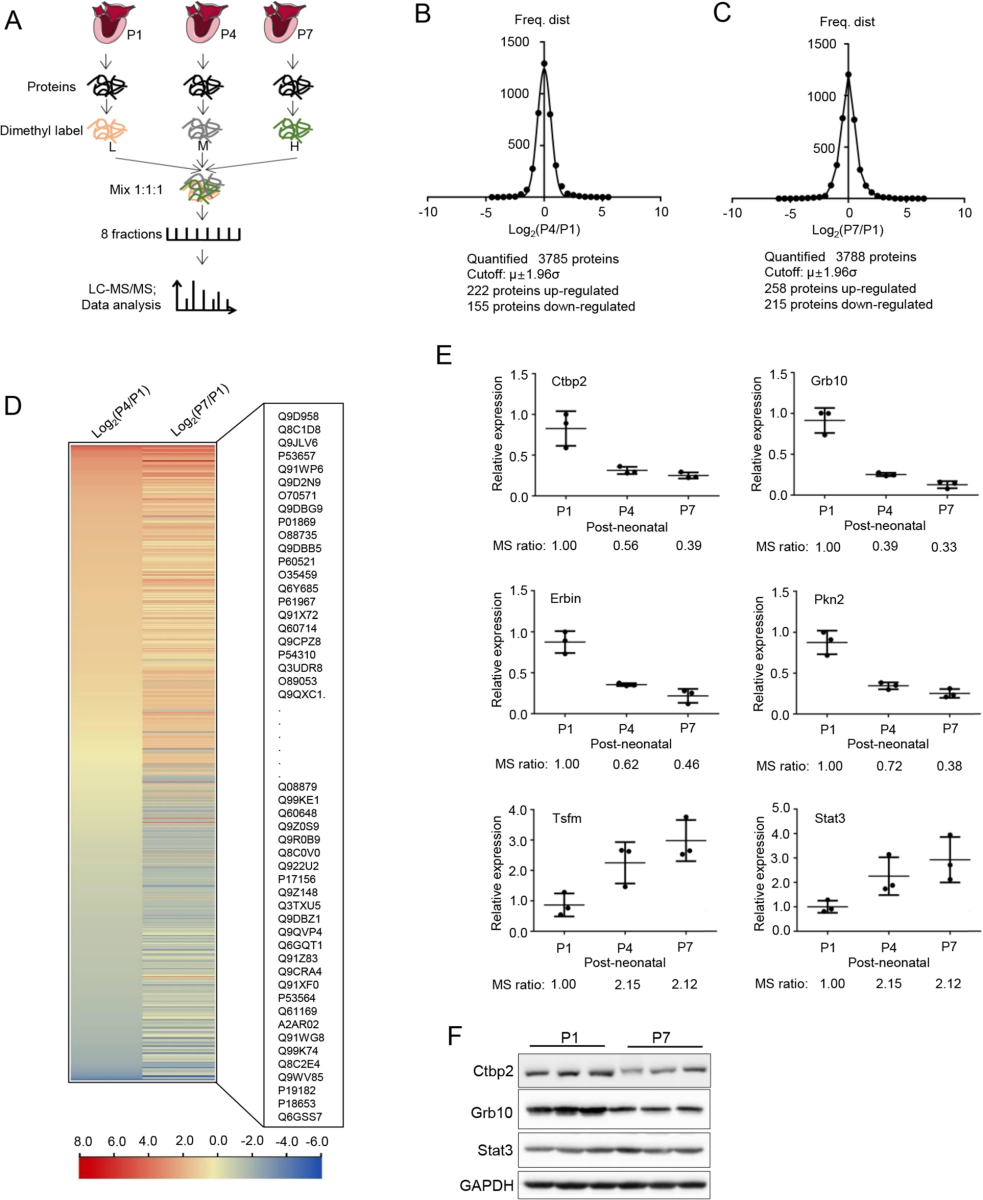
Age-related proteomic changes in neonatal C57BL/6J mouse hearts from day 1 to day 7. **A** Workflow of the quantitative proteomic analysis of neonatal mouse hearts; (**B**) and (**C**) Gaussian distribution of dimethyl labeling ratio. The “significantly changed” threshold was set by μ ± 1.96σ. The “significantly changed” ratios between the P4/P1 groups were > 2.18 or < 0.46, and between the P7/P1 groups > 2.27 or < 0.43; (D) Heatmap of significantly changed proteins. Red and blue reflect high and low expression, respectively; (E) Real-time PCR results of selected genes (*n* = 3). (F) Western blotting of selected genes (*n* = 3)

To determine the proteomic changes in neonatal C57BL/6 J mouse hearts from days 1–7, we conducted a quantitative proteomic analysis using neonatal mouse hearts. All neonatal mice were from the same families. As shown in Fig. 1A, mouse hearts from days 1 (P1 group), 4 (P4 group), and 7 (P7 group) were excised and the total protein was extracted. After protein digestion, dimethyl labeling, and SCX separation, the samples were analyzed using LC–MS/MS. Figure 1B shows the Gaussian distribution of the dimethyl-labeling ratio. For the P1 and P4 groups, a total of 3785 mouse proteins were quantified. The results showed that 222 proteins were upregulated and 155 proteins were downregulated in the P4 group compared with those in the P1 group (Fig. 1B). In the P1 and P7 groups, 3788 proteins were quantified, of which 258 were upregulated and 215 were downregulated in the P7 group compared with those in the P1 group (Fig. 1C). The expression levels of these differentially expressed proteins are shown in detail and visualized using heatmaps (Fig. 1D). As we focused on the myocardial development, we selected three genes related to body development (including CTBP2, STATA3, GRB10) and three genes that have not been confirmed to be related to development (PKN2, ERBIN, TSFM) to verify the proteomics results using real-time PCR assays (Fig. 1E). Three genes (Ctbp2, Grb10 and Stat3) were chosen to validate using western blot (Fig. 1F). The results showed that changes in RNA levels of all genes were consistent with those from MS quantification.

### Statistical enrichment test of mice heart

To determine whether a particular class of proteins was randomly distributed or enriched relative to the overall protein amount, a statistical enrichment test was used to assess the quantified proteins. All proteins were classified into 11 functional groups, including cell cycle, DNA packaging, DNA repair, ribosome biogenesis, cellular respiration, lipid metabolic process, ATP metabolic process, mitochondrial organization, cellular calcium ion homeostasis, phagocytosis, and inflammatory response. As shown in Figures S1 and S2, compared with the P1 group, the downregulated genes in the hearts of both P4 and P7 groups were mainly related to cell cycle, DNA packaging, DNA repair, and ribosome biogenesis. Moreover, the upregulated genes in the hearts of both P4 and P7 groups were mainly related to cellular respiration, lipid metabolic process, ATP metabolic process, mitochondrial organization, cellular calcium ion homeostasis, phagocytosis, and inflammatory response (Figure S1 and Figure S2).

### KEGG pathway analysis in mice heart

**Fig. 2:**
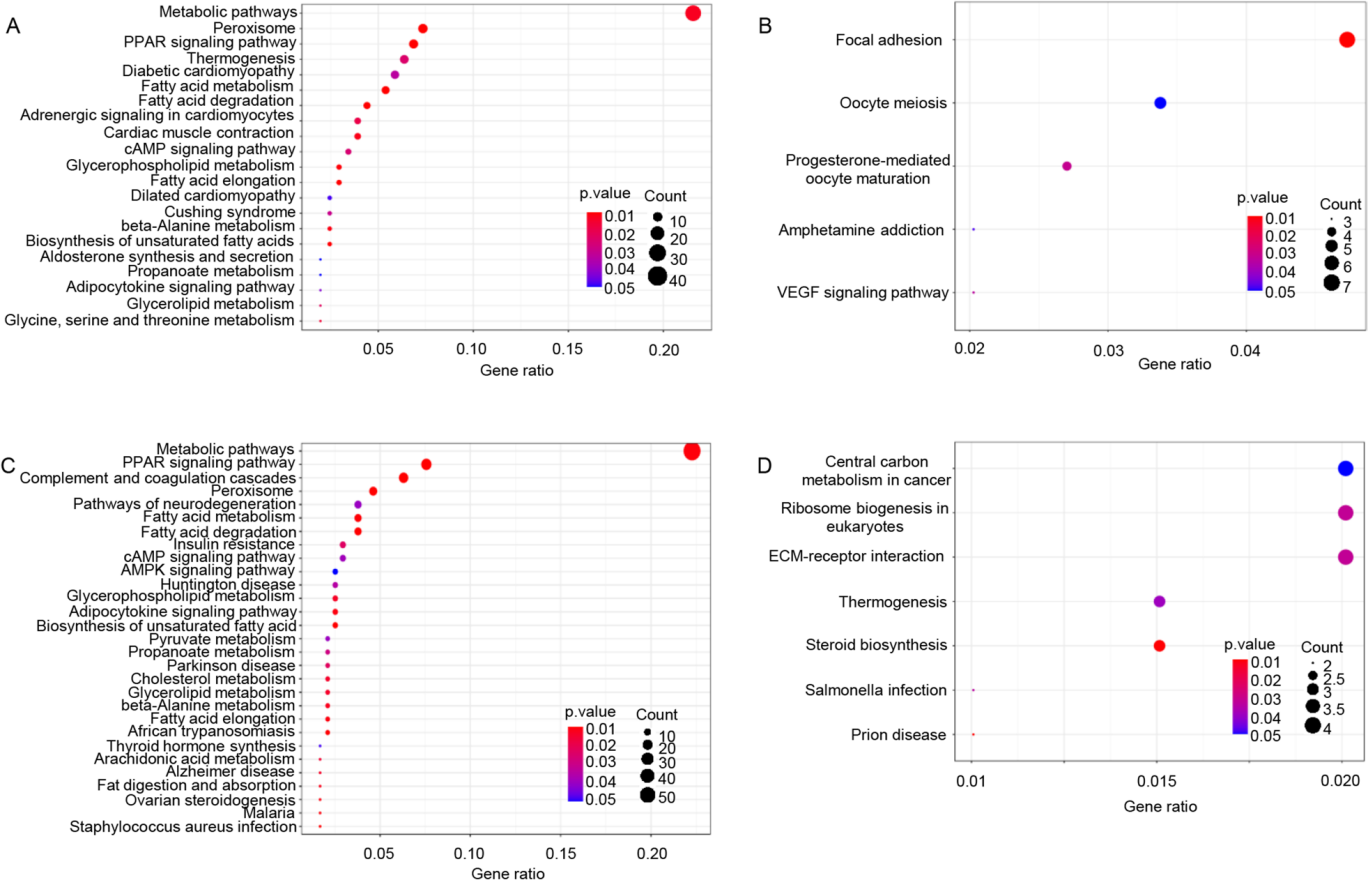
KEGG pathway analysis of significantly changed proteins. **A** Upregulated proteins enriched in KEGG pathways at day 4; **B** Downregulated proteins enriched in KEGG pathways at day 4; **C** Upregulated proteins enriched in KEGG pathways at day 7; **D** Downregulated proteins enriched in KEGG pathways at day 7

**Fig. 3:**
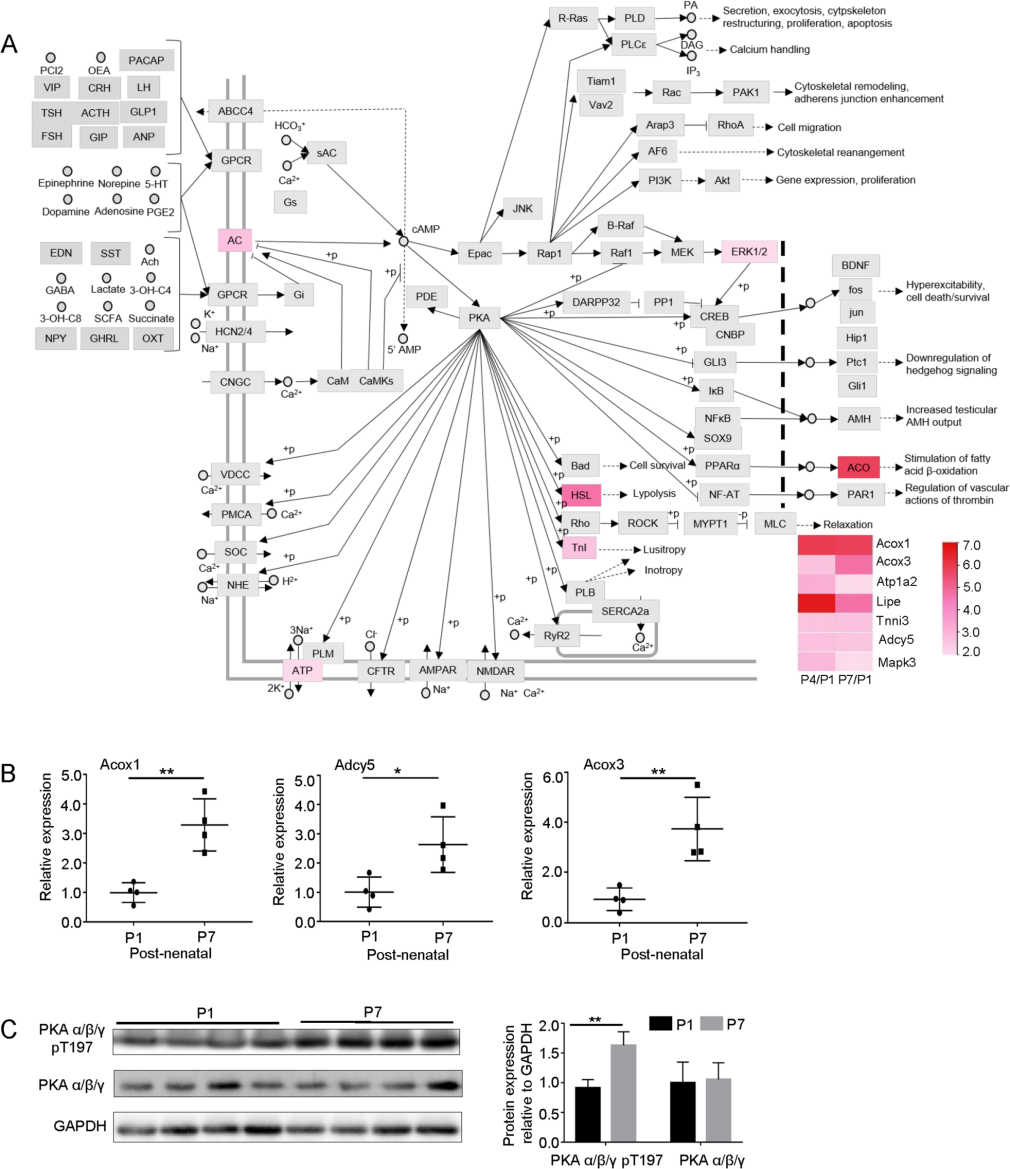
Validation of the cAMP signaling pathway-related proteomics results using PCR and western blotting. **A** Schematic representation of the cAMP signaling pathway; 7 upregulated genes are highlighted in red. Heatmap showing age-related changes in the expression levels of the seven genes; **B** RT-PCR results of the selected three genes from the altered seven genes (*n* = 4). **C** Western blotting of phosphorylated PKA and total PKA (*n* = 4)

Proteins exert their biological functions by participating in a series of biochemical reactions. Pathway analysis is a common approach to understanding these biological processes. Our findings showed that compared with P1 group, the upregulated proteins were mainly associated with “Peroxisome,” “PPAR signaling pathway,” and “cAMP signaling pathway” (Fig. 2A and C). Consistent with the PANTHER analysis, the upregulated proteins were also enriched in pathways such as “Fatty acid metabolism,” “Fatty acid degradation,” “Fatty acid elongation,” “Biosynthesis of unsaturated fatty acids,” “Glycerolipid metabolism,” “Cholesterol metabolism,” and “Fat digestion and absorption” in the hearts of P4 and P7 group (Fig. 3A and C). On the other hand, downregulated proteins pathways were enriched in “Focal adhesion,” “Oocyte meiosis,” “Progesterone-mediated oocyte maturation,” “Amphetamine addiction,” and “VEGF signaling pathway” in the hearts of the P4 group (Fig. 2B). The downregulated proteins were enriched in “Central carbon metabolism in cancer,” “Ribosome biogenesis in eukaryotes,” “ECM-receptor interaction,” “Thermogenesis,” “Steroid biosynthesis,” “*Salmonella* infection,” and “Prion disease” in the hearts of the P7 group (Fig. 2D).

### Role of the cAMP/PKA signaling pathway in cardiomyocyte replication

As the upregulated proteins were enriched in the “cAMP signaling pathway,” which was consistent in both groups P4 and P7 (Fig. 2A and C), we further explored the role of the cAMP signaling pathway in cardiomyocyte replication. Figure 3A shows the schematic representation of the cAMP signaling pathway. We found that seven genes in this pathway, including *Acox1, Acox3, apt1a1, Lipe, Tnni3, Adcy5*, and *Mapk3,* were upregulated in the hearts of the P4 and P7 groups (Fig. 3A), indicating the age-related activation of the cAMP pathway in the mouse myocardium. As PKA is the best described target of cAMP, we explored the role of PKA in cardiomyocyte replication.

Phosphorylation of Thr-197 (T197) during PKA activation is an important step for its biological function [27]. To study whether the cAMP/PKA signaling pathway had an age-related activation, RT-PCR assay of PKA-associated genes was performed using mouse heart samples from the P1 and P7 groups. The results showed that RNA levels of the three genes involved in cAMP signaling (*Acox1, Acox3* and *Adcy5*) were significantly higher in the P7 than in the P1 group (Fig. 3B). Consistent with the findings of the MS/MS quantification assay, western blotting indicated that there was increased phosphorylation of PKA at T197 in the P7 group compared with the P1 group (Fig. 3C).

**Fig. 4:**
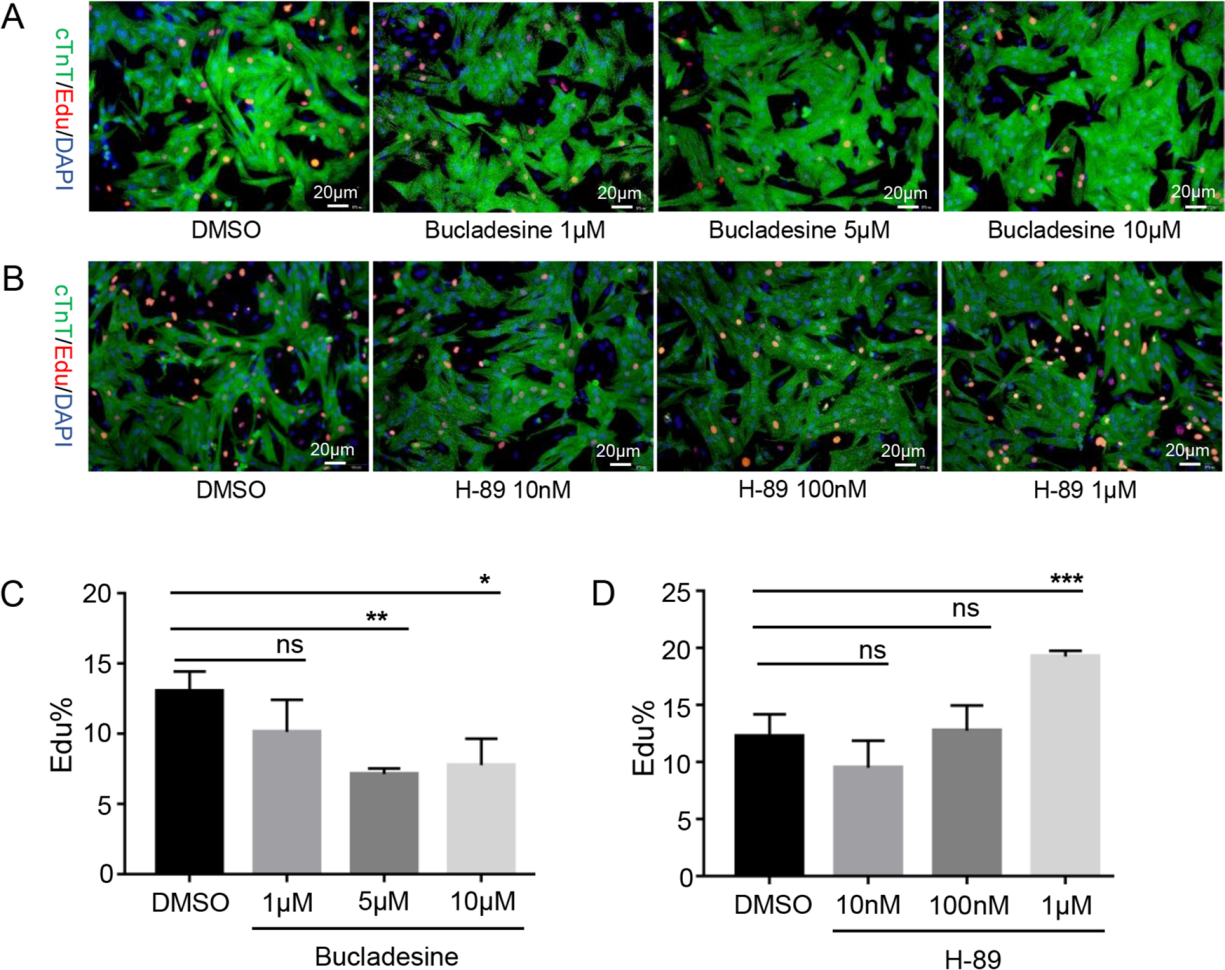
Inhibition of PKA promoted EdU-DNA incorporation in cardiomyocytes. **A**, **B** EdU-DNA incorporation assay in primary cardiomyocytes treated with DMSO and the PKA activator (1μM, 5μM, 10μM bucladesine) or the PKA inhibitor (10nM, 100nM, 1 μM H-89). EdU staining (red fluorescence), DAPI staining (blue fluorescence), scale bar = 20 μm; **C**, **D** Statistical analysis of EdU results. 5μM and 10μM bucladesine can inhibit cardiomyocyte replication (from 13.03% to 7.13% *p* = 0.0023 and 7.78% *p* = 0.0102) and 1μM H-89 can promote cardiomyocyte replication (from 12.26% to 19.25%, *p* = 0.0004)

**Fig. 5:**
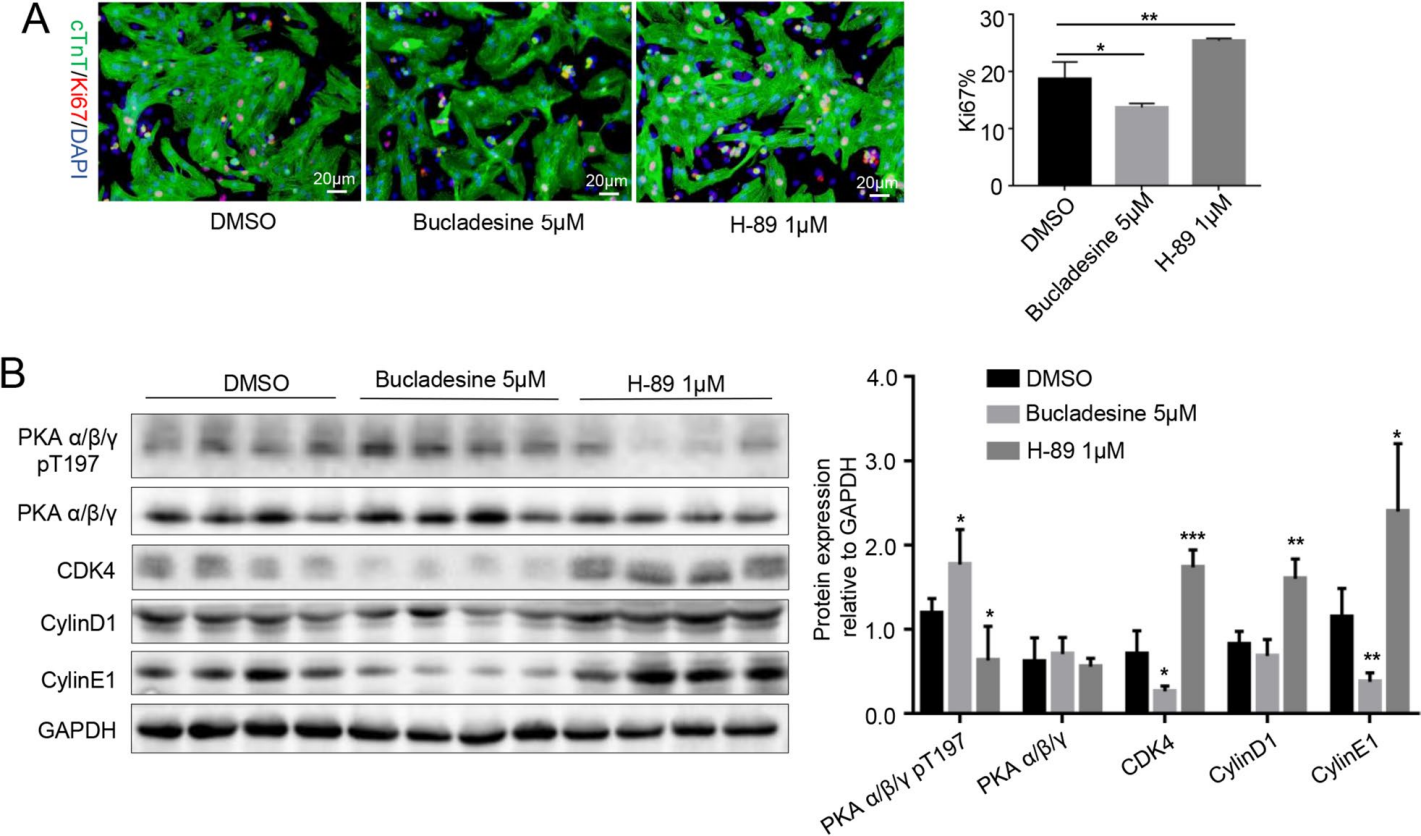
Inhibition of PKA-promoted cardiomyocyte replication and upregulated cell cycle–related genes. **A** Ki67 results of PKA inhibitor and activator; 5μM bucladesine inhibited cardiomyocyte replication (from 15.3% to 9.5%, *p* = 0.0047) and 1μM H-89 promoted cardiomyocyte replication (from 15.3% to 24.4%, *p* = 0.0017). **B** Western blotting results of PKA α/β/γ pT197, PKA α/β/γ, CDK4, CylinD1, and CylinE1

To further explore the role of the PKA pathway in cardiomyocytes, a PKA activator and inhibitor were used to stimulate neonatal mouse cardiomyocytes. We found that treatment with 5 μM and 10 μM of the PKA activator bucladesine could inhibit cardiomyocyte DNA synthesis (EdU^+^ from 13.03% to 7.13%, p = 0.0023; 7.78%, p = 0.0102) (Fig. 4A and C), whereas treatment with 1 μM of the PKA inhibitor H-89 promoted cardiomyocyte DNA synthesis (EdU^+^ cardiomyocytes from 12.26% to 19.25%, p = 0.0004) (Fig. 4B and D). As EdU only reflects DNA synthesis, we detected Ki67 expression, which is expressed in the G1, S, G2, and M phases of cell division. The results indicated that 5 μM bucladesine could inhibit cardiomyocyte replication (from 15.3% to 9.5%, p = 0.0047), whereas 1 μM H-89 could promote cardiomyocyte replication (from 15.3% to 24.4%, p = 0.0017) (Fig. 5A). Western blotting results were consistent with the Edu and Ki67 results. Thus, bucladesine treatment increased PKA phosphorylation at T197 and slightly downregulated the cell cycle-related proteins including CDK4, and CylinE1, whereas H-89 treatment decreased PKA phosphorylation level at T197 and promoted the expression of CDK4, CylinD1, and CylinE1 (Fig. 5B).

## Discussion

Heart disease is the leading cause of death worldwide. Heart muscle cells in the adult heart lack regenerative capacity and, thus, damaged necrotic heart muscle cells are replaced by fibrous tissue, leading to decreased heart function [2]. The underlying molecular mechanism of cardiac regeneration in newborn mice is still unknown, but it is expected to be caused by changes in gene and protein expression. Therefore, the systematic study of proteomic changes between regenerative and nonregenerative hearts is essential in understanding the molecular mechanism of cardiac regeneration.

Previous studies show that adult cardiomyocytes are generally highly differentiated, and damage to the hearts of mice older than a week is difficult to repair [28]. In this study, we compared the proteomes of neonatal mouse hearts at day 1, day 4, and day 7. The MS/MS analysis was carried out using a standard method and the findings were verified by RT-PCR and western blotting. It was also found that the functional PKA analysis was consistent with the proteomics results. Both the proteomic results and signaling pathway analysis demonstrated that activation of cAMP signaling was age-related and increased in mouse hearts. Furthermore, the cAMP signaling pathway was involved in regulating cardiomyocyte replication. As far as we know, this is the first study showing that activation of the cAMP signaling pathway inhibited myocardial cells from entering the cell cycle.

The recent development of comparative proteomics technology has provided a technical platform to study the molecular mechanisms underlying myocardial regeneration, allowing the rapid accumulation of data on myocardial regeneration-related and signaling pathway–related factors [29]. This helps researchers explain the mechanism of a certain signaling pathway and further understand its position in the overall regulatory network of myocardial regeneration. In this study, we used LC–MS/MS proteomic analysis to determine the differences in neonatal mouse hearts at days 1, 4, and 7. KEGG pathway analysis showed changes in many protein groups including those in the cAMP signaling pathway.

Previous studies have shown that protein kinases regulate various cellular functions and as potential therapeutic targets for the management of cardiovascular diseases [30]. As an important signal transduction factor, PKA plays a key role in various cell regulation processes by phosphorylating target proteins and altering their activity. The basal activity and activation of PKA are considered to be closely related to cardiac diseases [31]. In this study, we found that the PKA activator could inhibit cardiac regeneration and that the PKA inhibitor could promote cardiomyocyte proliferation. Furthermore, we explored the mechanism of PKA in inhibiting cardiomyocyte proliferation. Results from western blot suggested an increase in the protein levels of cell cycle-related genes in PKA activator treated-cardiomyocytes. These data indicate that PKA kinase may play a crucial role in neonatal myocardial regeneration, that is, modulating the proteins related to cell proliferation.

## Conclusions

In this study, we conducted a quantitative proteomic analysis of neonatal mouse hearts at day 1, day 4, and day 7. Comparison of the P4 group with the P1 group, a total of 3785 proteins were quantified of which 222 proteins were upregulated and 155 proteins were downregulated. Comparison of the P1 and P7 groups led to quantification of 3788 proteins, of which 258 were upregulated and 215 were downregulated in the P7 group compared with those in the P1 group. Bioinformatic analysis showed that proteins related to the cell cycle, DNA packaging, DNA repair, and ribosome biogenesis were significantly downregulated in correspondence with the development of the neonatal mouse heart, while proteins involved in cellular respiration, lipid metabolic process, ATP metabolic process, mitochondrion organization, cellular calcium ion homeostasis, phagocytosis, and the inflammatory response are upregulated from day 1 to day 7. KEGG pathway analysis showed changes in many protein groups, including those associated with the PKA pathway. Further study suggested that PKA activity was closely related to cardiomyocyte replication. Treatment with the PKA inhibitor could promote cardiomyocyte division, whereas treatment with the PKA agonist inhibited cardiomyocyte entry into the cell cycle. These results indicated that PKA may play a critical role in neonatal myocardial regeneration. These findings provide a new experimental foundation for pathological cardiac regeneration, suggesting novel treatment strategies.

### Supplementary Information

**Additional file 1:** **Figure S1.** Statistical enrichment test” results from the PANTHER website. Changes in protein enrichment between the P1 and P4 groups determined by the “statistical enrichment test.

**Additional file 2:** **Figure S2.** Statistical enrichment test” results from the PANTHER website. Changes in protein enrichment between the P1 and P7 groups determined by the “statistical enrichment test.

**Additional file 3.**

## Data Availability

The data that support the findings of this study are available in the figshare database (https://figshare.com/s/87e7efcc4e0f1aa40a95).
