# Supplementary figures and images for: Proteomic analysis of neonatal mouse hearts shows PKA functions as a cardiomyocyte replication regulator
Source: Proteome Sci. 2023 Oct 11;21:16. doi: 10.1186/s12953-023-00219-4 (PMC10566114; doi:10.1186/s12953-023-00219-4)

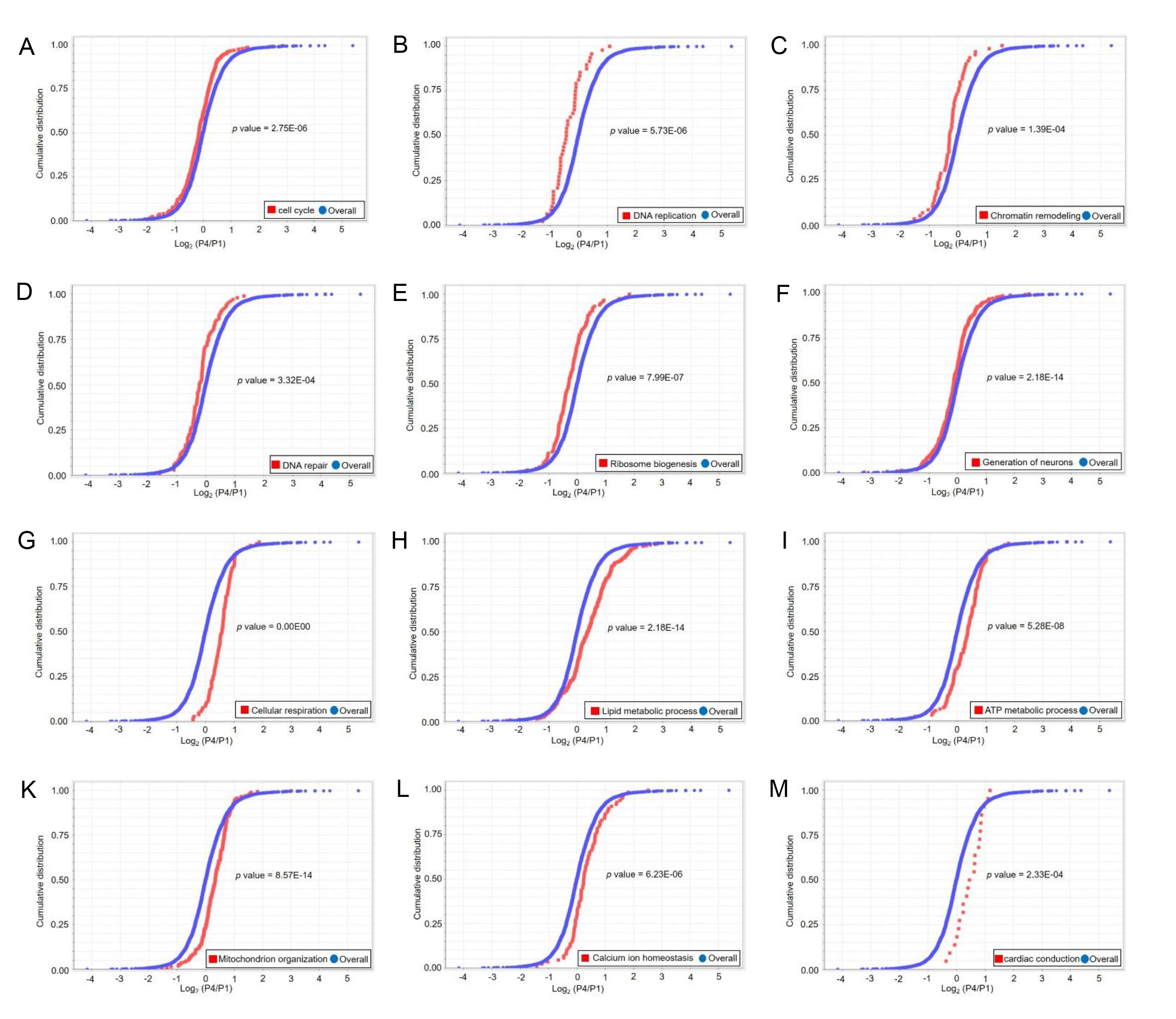

Supplement: Supplementary file 1 — Additional file 1: Figure S1. Statistical enrichment test” results from the PANTHER website. Changes in protein enrichment between the P1 and P4 groups determined by the “statistical enrichment test. [file 12953_2023_219_MOESM1_ESM.tif]

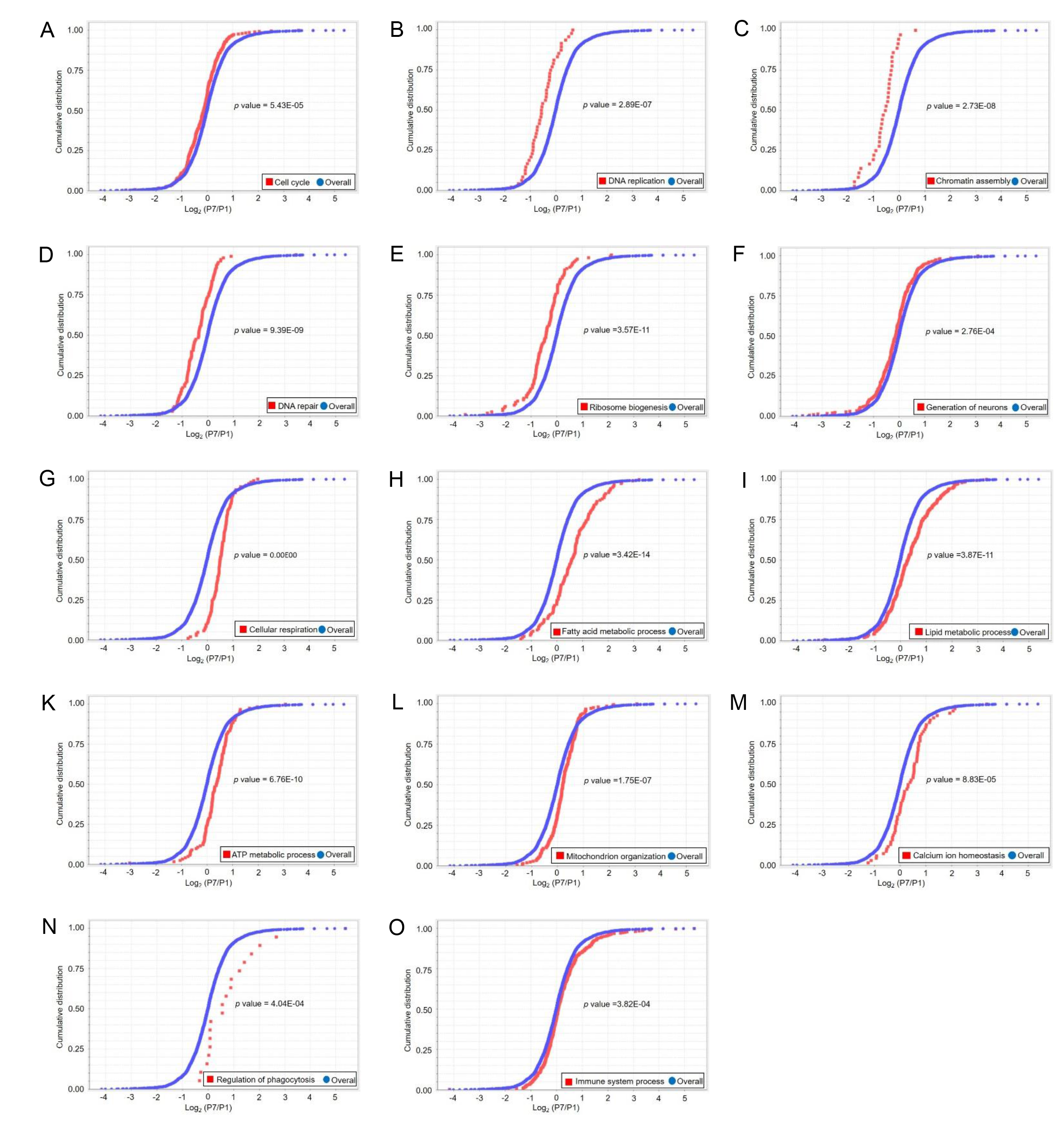

Supplement: Supplementary file 2 — Additional file 2: Figure S2. Statistical enrichment test” results from the PANTHER website. Changes in protein enrichment between the P1 and P7 groups determined by the “statistical enrichment test. [file 12953_2023_219_MOESM2_ESM.tif]
