# Supplementary material for: Proteomic analysis of neonatal mouse hearts shows PKA functions as a cardiomyocyte replication regulator
Source: Proteome Sci. 2023 Oct 11;21:16. doi: 10.1186/s12953-023-00219-4 (PMC10566114; doi:10.1186/s12953-023-00219-4)

Figure 1. Age-related proteomic changes in neonatal C57BL/6J mouse hearts from day 1 to day 7. (F) Western blotting of selected genes (n=3).

Figure 1F Ctbp2

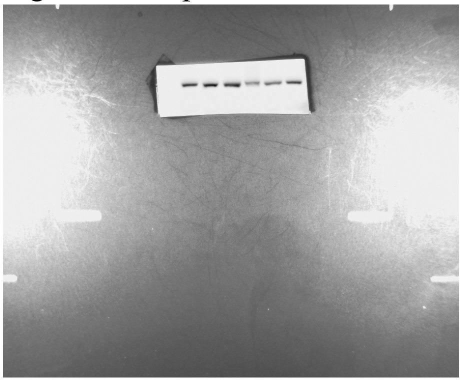

Figure 1F Grb10

Figure 1F Stat3

Figure 1F GAPDH

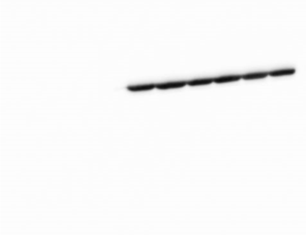

Figure 3. Validation of the cAMP signaling pathway-related proteomics results using PCR and western blotting. C. Western blotting of phosphorylated PKA and total PKA (n=4).

Figure 3C PKA α/β/γ pT197

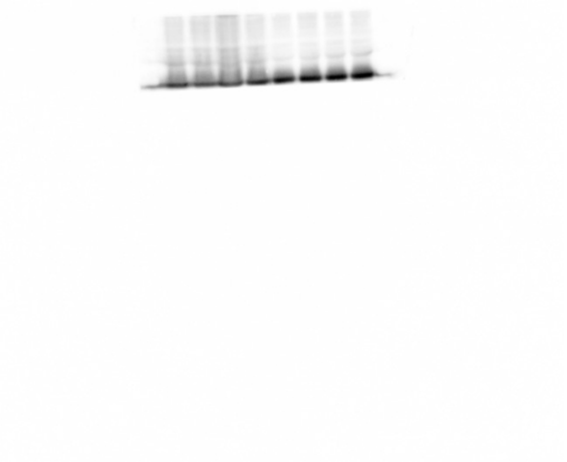

Figure 3C PKAα/β/γ

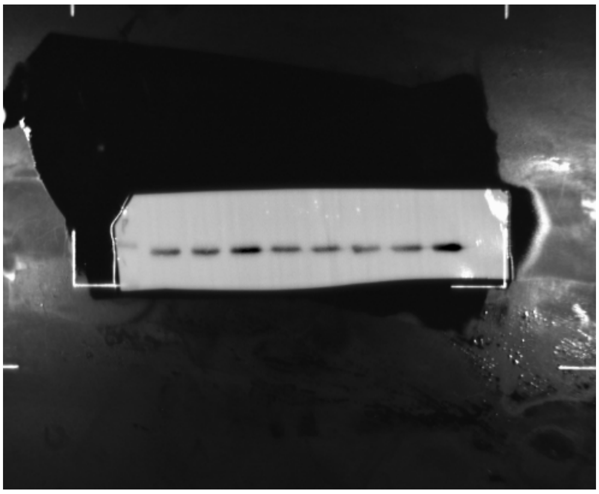

Figure 3C GAPDH

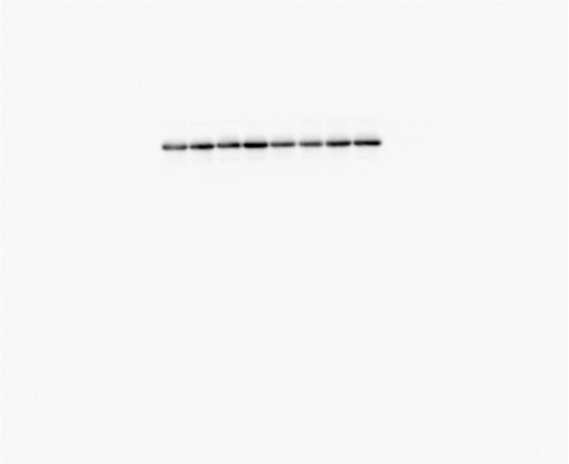

Figure 5. Inhibition of PKA-promoted cardiomyocyte replication and upregulated cell cycle–related genes. B. Western blotting results of PKA α/β/γ pT197, PKA α/β/γ, CDK4, CylinD1, and CylinE1.

Figure 5B PKA α/β/γ pT197

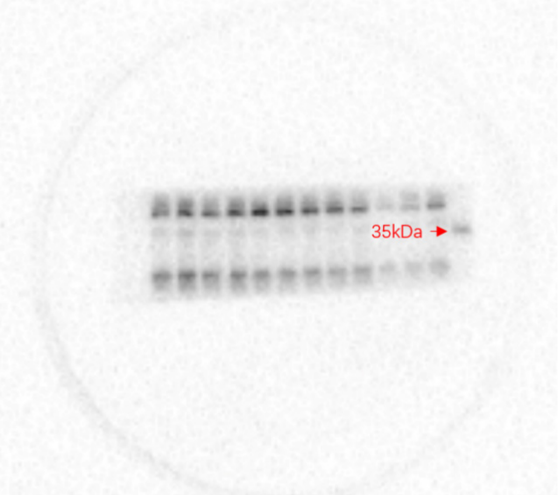

Figure 5B PKAα/β/γ

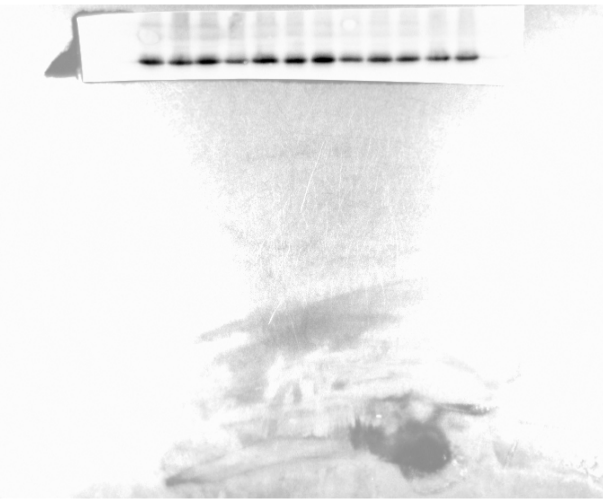

Figure 5B CDK4

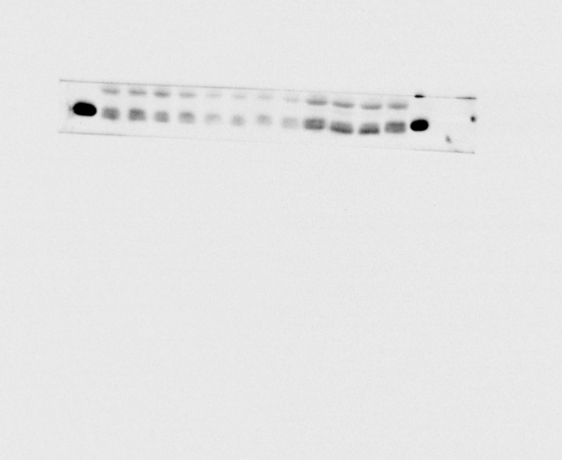

Figure 5B CylinD1

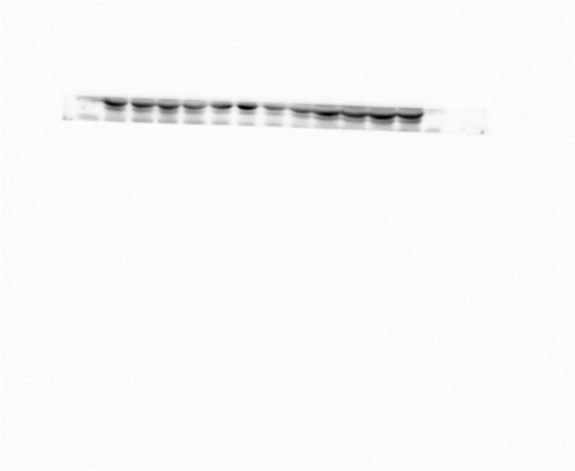

Figure 5B CylinE1

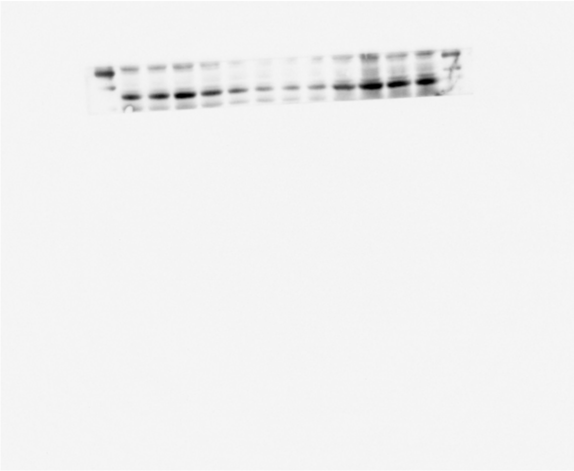

Figure 5B GAPDH

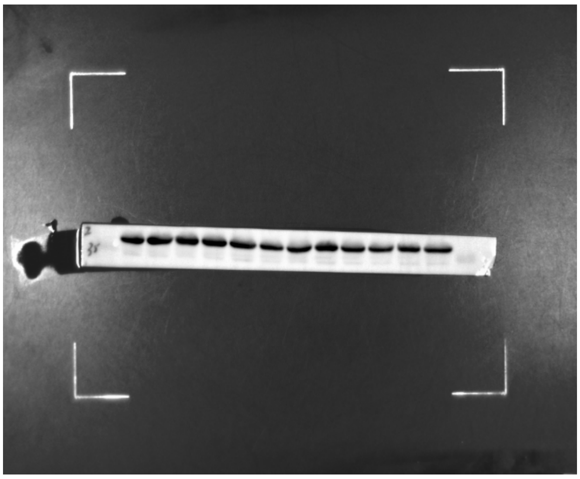

Supplement: Supplementary file 3 — Additional file 3. [file 12953_2023_219_MOESM3_ESM.pdf]
